# Supplementary material for: Impact of Inbreeding and Ancestral Inbreeding on Longevity Traits in German Brown Cows
Source: Animals (Basel). 2023 Aug 30;13(17):2765. doi: 10.3390/ani13172765 (PMC10486702; doi:10.3390/ani13172765)
Supplement: Supplementary file 1 [file animals-13-02765-s001.zip › Supplementary Table S1.pdf]

Supplementary Table S1: Means, standard deviations (SD), minima, maxima of classical inbreeding (F), inbreeding coefficient according to Ballou (1997) (F_{a_Bal}), ancestral history coefficient according to Baumung (2005) (Ahc), ancient (F_{a_Kal}) and new (F_{New}) inbreeding coefficient according to Kalinowski (2002) for German Brown born between 1990 and 2001.

	Mean	SD	Min	Max
F	0.018	0.018	0	0.317
F_{a_Ba}l	0.007	0.012	0	0.181
Ahc	0.007	0.012	0	0.189
F_{a_Kal}	0.0003	0.001	0	0.066
F_{New}	0.007	0.014	0	0.253

Supplementary Table S2: Pearson correlation coefficients of inbreeding coefficients, heterosis and recombination coefficients for German Brown cows born between 1990 and 2001.

	F_{a_Bal}	Ahc	F_{a_Kal}	F_{New}	HET	REC
F	0.074	0.074	0.043	0.046	-0.329	0.026
F_{a_Bal}		1.000	0.481	0.255	-0.108	-0.086
Ahc			0.559	0.254	-0.107	-0.086
F_{a_Kal}				0.481	-0.063	-0.057
F_{New}					-0.067	-0.046
HET						0.157
REC						

Supplementary Table S3. Inbreeding depression per 1% inbreeding expressed in percentage of the respective trait mean ($\bar{x}$), phenotypic (σ_P) and genetic (σ_a) standard deviation.

	$\bar{x}$	σ_P	σ_a
HL (years)	-0.32	-0.91	-2.27
LPL (years)	-0.61	-0.97	-2.42
NC	-0.71	-1.19	-2.99
LMY (kg)	-0.83	-1.27	-10.98
LFY (kg)	-0.85	-1.32	-3.71
LPY (kg)	-0.84	-1.29	-3.61
EffLMY (kg/day)	-0.465	-1.416	-3.610
EffLFY (kg/day)	-0.483	-1.504	-4.198
EffLPY (kg/day)	-0.465	-1.426	-3.853
Surv1	-0.343	-0.729	-4.581
Surv3	-1.065	-1.015	-3.847
Surv5	-1.953	-0.991	-3.345
Surv7	-2.333	-0.625	-2.279
Surv9	-3.059	-0.405	-2.207
Cul_{CL}	-0.822	-0.300	-1.275
Cul_{INF}	1.583	0.943	5.129
Cul_{UD}	-0.087	-0.391	-2.044

* F-1% = F/100, inbreeding depression by trait mean ($\bar{x}$) = (F-1%/$\bar{x}$)*100, inbreeding depression by phenotypic standard deviation (σ_P) = (F-1%/σ_P)*100, inbreeding depression by genetic standard deviation (σ_a) = (F-1%/σ_a)*100

Supplementary Table S4. Expected effects of heterosis, recombination and inbreeding for longevity traits in German Brown cows analysed (birth years 1990 to 2001) and projected for German Brown cows of the birth year 2014

Trait	Analysed population				Projected for 2014 birth year population			
	HET	REC	F	Combined effects	HET	REC	F	Combined effects
HL (years)	0.192	0.059	-0.036	0.215	0.167	0.055	-0.074	0.147
LPL (years)	0.185	0.060	-0.038	0.207	0.160	0.056	-0.079	0.137
NC	0.137	0.048	-0.045	0.140	0.119	0.044	-0.093	0.071
LMY (kg)	749.857	137.961	-334.014	553.805	652.087	128.016	-692.150	87.953
LFY (kg)	29.443	7.350	-14.233	22.561	25.604	6.821	-29.493	2.932
LPY (kg)	22.286	3.692	-11.985	13.993	19.381	3.426	-24.835	-2.029
Eff_LMY (kg/day HL)	0.028	-0.047	-0.070	-0.089	0.025	-0.044	-0.146	-0.165
Eff_LFY (kg/day HL)	0.000	-0.002	-0.003	-0.005	0.000	-0.002	-0.006	-0.008
Eff_LPY (kg/day HL)	-0.001	-0.002	-0.003	-0.006	-0.001	-0.002	-0.005	-0.008
Surv1	0.015	0.008	-0.005	0.018	0.013	0.007	-0.010	0.011
Surv3	0.030	0.014	-0.008	0.035	0.026	0.013	-0.017	0.022
Surv5	0.026	0.011	-0.007	0.030	0.023	0.011	-0.014	0.019
Surv7	0.011	0.006	-0.003	0.014	0.010	0.005	-0.006	0.009
Surv9	0.004	-0.001	-0.001	0.002	0.003	-0.001	-0.002	0.001
Cul_{CL}	-0.004	-0.015	-0.002	-0.021	-0.004	-0.014	-0.004	-0.021
Cul_{INF}	0.015	0.013	0.007	0.036	0.013	0.012	0.015	0.040
Cul_{UD}	-0.002	-0.004	-0.002	-0.007	-0.001	-0.003	-0.005	-0.009

Means of heterosis = 0.441, recombination = 0.369, and inbreeding coefficient = 0.018 of the German Brown cows born between 1990 and 2001 and corresponding values of cows born in 2014 for heterosis, recombination and inbreeding coefficient are 0.3835, 0.3424, and 0.0373, respectively.

Effects of HET, REC and F were calculated by multiplying the results of Model 1 (Table 3) with the mean coefficient of heterosis, recombination and inbreeding, respectively.

Combined effects are calculated as sum of the expectations for heterosis, recombination and inbreeding effects.

Supplementary Table S5a. Expected phenotypes and differences between cows with low (5% percentile) and high (95% percentile) degree of inbreeding and differences between fully inbred and non-inbred cows for the classical inbreeding coefficient F.

	High (95%)	Low (5%)	Difference High 95% - Low 5%	Difference 100% - 0%
HL (years)	6.09	6.19	0.09	0.63
LPL (years)	3.40	3.50	0.10	0.67
NC	3.44	3.55	0.12	0.79
LMY (kg)	21779.66	22652.92	873.26	5891.44
LFY (kg)	910.02	947.23	37.21	251.04
LPY (kg)	777.65	808.98	31.33	211.39
EffLMY (kg/day)	8.31	8.49	0.18	1.24
EffLFY (kg/day)	0.347	0.355	0.008	0.05
EffLPY (kg/day)	0.297	0.304	0.007	0.04
Surv1	0.752	0.765	0.012	0.08
Surv3	0.416	0.437	0.022	0.15
Surv5	0.182	0.200	0.018	0.12
Surv7	0.062	0.069	0.007	0.05
Surv9	0.015	0.018	0.002	0.02
Cul_{CL}	0.115	0.120	0.005	0.03
Cul_{INF}	0.264	0.245	-0.019	-0.13
Cul_{UD}	0.111	0.117	0.006	0.04

F (5% percentile) = 0; F (95% percentile) = 0.047.

Supplementary Table S5b. Expected phenotypes of low (5% percentile) and high (95% percentile) inbred cows for F_{a_Kal}, F_{New} and Ahc.

	F_{a_Kal}			F_{New}			Ahc		
	High	Low	Difference	High	Low	Difference	High	Low	Difference
HL (years)	**6.14**	**6.15**	**0.01**	6.150	6.150	0.000	**6.13**	**6.16**	**0.03**
LPL (years)	**3.45**	**3.46**	**0.01**	3.460	3.460	0.000	**3.44**	**3.47**	**0.03**
NC	**3.50**	**3.51**	**0.01**	3.510	3.510	0.000	**3.49**	**3.52**	**0.02**
LMY (kg)	**22248.84**	**22333.94**	**85.11**	22318.39	22319.18	0.80	**22202.03**	**22354.34**	**152.31**
LFY (kg)	**930.22**	**933.59**	**3.37**	932.98	933.01	0.03	**928.94**	**934.23**	**5.29**
LPY (kg)	**794.53**	**797.53**	**2.99**	796.98	797.01	0.03	**793.15**	**798.16**	**5.01**
EffLMY (kg/day)	8.408	8.423	0.015	8.42	8.42	0.00	**8.404**	**8.425**	**0.02**
EffLFY (kg/day)	0.352	0.352	0.001	0.35	0.35	0.00	0.352	0.352	0.001
EffLPY (kg/day)	0.301	0.301	0.001	0.30	0.30	0.00	0.301	0.301	0.001
Surv1	0.760	0.760	0.000	0.76	0.76	0.00	0.759	0.760	0.001
Surv3	0.426	0.430	0.003	0.43	0.43	0.00	0.426	0.430	0.004
Surv5	**0.191**	**0.193**	**0.002**	0.19	0.19	0.00	0.191	0.194	0.003
Surv7	0.066	0.066	0.001	0.07	0.07	0.00	0.065	0.066	0.002
Surv9	0.016	0.017	0.001	0.02	0.02	0.00	0.016	0.017	0.001
Cul_{CL}	0.118	0.118	0.000	0.12	0.12	0.00	**0.118**	**0.118**	**0.000**
Cul_{INF}	0.252	0.252	0.000	0.25	0.25	0.00	**0.252**	**0.252**	**0.000**
Cul_{UD}	0.116	0.115	-0.001	**0.12**	**0.11**	**0.00**	0.116	0.115	-0.001

F_{a_Kal}, F_{New}, Ahc (5% percentile) = 0; F_{a_Kal} (95% percentile) = 0.002, F_{New} (95% percentile) =0.032, Ahc (95% percentile) = 0.031, numbers in bold type are based on significant regression coefficients.

Supplementary Table S5c. Observed inbreeding coefficients (F, F_{a_Kal}, F_{New} and Ahc) for survival to 2nd (Surv1), 4th (Surv3), 6th (Surv5), 8th (Surv7), and 10th (Surv9) lactation number and culling rate due to foot and leg problems (Cul_{CL}), infertility (Cul_{INF}) and udder diseases (Cul_{UD}).

Inbreeding coefficient	Surv1		Surv3		Surv5		Surv7		Surv9	
	No	Yes	No	Yes	No	Yes	No	Yes	No	Yes
F	0.0188750	0.0177215	0.0185457	0.0172693	0.0182935	0.0167630	0.0181203	0.0162772	0.0180385	0.0156681
F_{a_Ka}	0.0003728	0.0003675	0.0003738	0.0003620	0.0003707	0.0003605	0.0003696	0.0003568	0.0003694	0.0003319
F_{New}	0.0074388	0.0073201	0.0073898	0.0072937	0.0073625	0.0072906	0.0073601	0.0071859	0.0073557	0.0069312
F_{a_Bal}	0.0072461	0.0071495	0.0072213	0.0071079	0.0071909	0.0070966	0.0071851	0.0069973	0.0071780	0.0068622
Ahc	0.0073369	0.0072386	0.0073114	0.0071967	0.0072806	0.0071856	0.0072749	0.0070832	0.0072676	0.0069471

Inbreeding coefficient	Cul_{CL}		Cul_{INF}		Cul_{UD}	
	No	Yes	No	Yes	No	Yes
F	0.0180095	0.0179193	0.0179735	0.0180740	0.0180128	0.0178912
F_{a_Ka}	0.0003700	0.0003595	0.0003700	0.0003649	0.0003673	0.0003800
F_{New}	0.0073740	0.0071595	0.0073595	0.0073163	0.0073511	0.0073300
F_{a_Bal}	0.0071925	0.0070254	0.0071988	0.0070953	0.0071516	0.0073346
Ahc	0.0072822	0.0071132	0.0072889	0.0071831	0.0072408	0.0074267

Supplementary Table S6. Inbreeding coefficients of cows that survived the respective lactation number (1, 3, 5, 7, 9) compared with non-survivers (Surv1, Surv3, Surv5, Surv7, Surv9)

	F (survived)	SE	F (not survived)	SE	P-value
Surv1	0.0177	<0.0001	0.0189	<0.0001	<0.0001
Surv3	0.0173	<0.0001	0.0185	<0.0001	<0.0001
Surv5	0.0168	<0.0001	0.0183	<0.0001	<0.0001
Surv7	0.0163	<0.0001	0.0181	<0.0001	<0.0001
Surv9	0.0157	<0.0001	0.0180	<0.0001	<0.0001

Supplementary Table S7. Studies analyzing classical (F) and ancestral inbreeding coefficients (F_{a_Bal}, F_{a_Kal}, F_{New} and Ahc) with their means and standard deviations (in brackets) in Holstein dairy cattle.

Reference	Country	N_{PED}	N_A, N_R*	F	F_{a_Kal}	F_{New}	F_{a_Bal}	Ahc	Traits
Mc Parland et al. (2009) [23]	IRE	3.6 Mio	88,366	2.58-2.68	2.15-2.22	0.43-0.46			Milk, fertility, survival
Hinrichs et al. (2015) [7]	GER	73,946	36,477	2.27	0.47	1.79	5.69		Birthweight, calving ease, stillbirth
Doekes et al. (2019) [6]	NL	167,924	38,792*	5.03 (1.81)	2.73 (0.91)	2.29 (1.08)		0.31 (0.05)	Milk, fertility, SCS
Makanjuola et al. (2020) [24]	CA	259,871	46,430*						Milk, fertility, SCS
Tohidi et al. (2023) [25]	IR	2.3 Mio	241,822	1.30 (1.88)	0.07 (0.16)	1.23 (1.23)		1.44 (.156)	Milk

IRE: Ireland, GER: Germany, NL: Netherlands, CA: Canada, IR: Iran; N_{PED}: number of animals in the pedigree; N_A: number of animals analyzed; N_R: Number of records; Milk: 305-day lactation records in first or first three lactations; SCS: somatic cell score in milk; Survival: survival from 1st to 2nd lactation number; Fertility: calving interval, age at first calving.
